# Cholesterol Metabolism in T Cells

**DOI:** 10.3389/fimmu.2017.01664

**Published:** 2017-11-27

**Authors:** Andreas Bietz, Hengyu Zhu, Manman Xue, Chenqi Xu

**Affiliations:** ^1^State Key Laboratory of Molecular Biology, Chinese Academy Center for Excellence in Molecular Cell Science, Shanghai Institute of Biochemistry and Cell Biology, Chinese Academy of Sciences, University of Chinese Academy of Sciences, Shanghai, China; ^2^University of Heidelberg, Heidelberg, Germany; ^3^School of Life Science and Technology, ShanghaiTech University, Shanghai, China

**Keywords:** cholesterol, metabolism, T cell, disease, immunomodulation

## Abstract

Compartmentalization and spatial control of biochemical reactions is the foundation of cell-based life on earth. The lipid bilayer system employed by eukaryote cells not only keeps them separate from the environment but also provides a platform for key receptors to sense and interact with outside factors. Arguably one of the cell types most reliant on interactions of this kind, immune cells depend on their membrane to keep functioning properly. In this review, the influence of variation in cholesterol levels, a key component of lipid bilayer stability, on T cells will be discussed in detail. In comparison to other cells, T cells must be able to undergo rapid activation followed by proliferation. Furthermore, receptor colocalization is an important mechanism in this activation process. The impact of cholesterol availability on the processes of T cell proliferation and receptor sensitivity, as well as its potential for immunomodulation in disease treatment will be considered.

## Cholesterol Metabolism

In the cell, cholesterol is largely responsible for being able to maintain a cell membrane with a certain degree of stiffness to it, removing the need for a rigid cell wall (1). This is due to its molecular tetracyclic structure, making it planar and rigid, as well as to the attached hydroxyl group which allows for orientation toward a polar solvent. It also serves as the substrate for a multitude of steroid-based hormones. Since it is so vital to every cell, the involved synthesis and regulatory pathways are nearly ubiquitous across cell populations (1).

### Biosynthesis

There are two major ways for a cell to acquire cholesterol: it can scavenge cholesterol from its surroundings (blood stream, etc.) or it can synthesize cholesterol itself. While a sizable amount of cholesterol is indeed ingested by most humans every day *via* animal tissue, the ratio of scavenged cholesterol vs. synthesized cholesterol favors the internally produced version (2). To preserve homeostasis, a transport mechanism from regions of high cholesterol to regions of low cholesterol, as well as regions where excess cholesterol is disposed of, must exist. Since cholesterol cannot be transported *via* the blood stream, it is bound up in clusters of lipids and proteins, either in the form of high-density lipoprotein (HDL) or low-density lipoprotein (LDL). Both categories have distinct regulatory mechanisms (2).

In the cell, cholesterol is synthesized from acetyl-coenzyme A, which is soon converted to hydroxymethylglutaryl-coenzyme A (HMG-CoA). This precursor goes through a multistep enzymatic reaction *via* intermediaries like mevalonate, squalene, and lanosterol until it eventually yields cholesterol. The biosynthesis is very well understood and documented by now. A less well-understood topic is the regulation of the metabolic pathway.

### Regulatory Mechanisms

The primary signaling molecule at work in the cholesterol pathway is without a doubt cholesterol itself. Synthesis of more cholesterol is regulated *via* a feedback mechanism. Exogenous cholesterol therefore also decreases synthesis and *vice versa*. The main regulatory mechanisms will be showcased based on signaling in either a low-oxysterol or a high-oxysterol state (3).

The key protein for cholesterol sensing is sterol response element-binding protein (SREBP). This protein is situated in the ER membrane, bound to a second protein called SREBP cleavage-activating protein (SCAP). Normally, these two proteins move from the ER and to the Golgi apparatus, where SREBP is processed further. SCAP functions as mediator of this transport (4).

In high-oxysterol situations, however, another protein called insulin-induced gene protein (INSIG) is present in the ER and binds to SCAP. This binding keeps the SREBP–SCAP pair rooted in the ER membrane. Only in a low-oxysterol environment does INSIG disassociate from SCAP, enabling further processing. Once transported to the Golgi apparatus, SREBP is cleaved by site-1 and 2 proteases (S1P and S2P), yielding its active transcription factor (TF) form (5). This TF fragment then migrates to the nucleus and modulates expression of enzymes responsible for the biosynthesis pathway (HMG-CoA synthase, HMG-CoA reductase, farnesyl diphosphate synthase, squalene synthase, etc.), chief among them the rate-limiting enzyme HMG-CoA reductase (1). This manifold expression profile is divided between the different variants of SREBP, such as SREBP1a, SREBP1c, and SREBP2. SREBP1c and SREBP2 can be roughly categorized as regulators of fatty acid metabolism and cholesterol metabolism, respectively (4). In addition, SREBP2 also mediates lipid transport and lipoprotein uptake. SREBP1a targets genes in both categories, also including transport. There is a certain degree of redundancy to the signaling of SREBP1 and 2, and it has previously been demonstrated that this redundancy makes it possible for dysregulation of one component to be compensated by the other.

Conversely, there is a TF limiting intracellular cholesterol levels: liver X receptor. This receptor forms a heterodimer with retinoid X receptor and binds cholesterol (and other oxysterols) as well as 9-cis retinoic acid; hence, it is active in a high-cholesterol environment. There are two isoforms of the receptor, LXRα and LXRβ with distinct expression profiles. While LXRα is primarily expressed in adipocyte and liver tissue, LXRβ is expressed more broadly, with enrichment in immune cells as well as heart and lungs. LXR has many downstream targets, most of them related to lipid metabolism. In reaction to sensing an excess of cholesterol, transferases such as cholesteryl ester transfer protein and ATB-binding cassette transporter A1 or G1 (ABCA1/ABCG1) mediate the efflux of cholesterol into HDL. Excess cholesterol is then transported to the liver either to be eventually excreted or converted to hormones and other molecules such as bile acid (6). The low-density lipoprotein receptor (LDLR) and the cholesterol esterification enzymes SOAT1/2 (alternative name ACAT1/2) also regulate intracellular cholesterol levels by changing the uptake rate or catalyzing conversion of cholesterol into cholesterol esters.

### Impact of Cholesterol Metabolism Defects

Recalling the emphasis on the importance of cholesterol to every cell in the body, it is clear, that defects in the system will be reflected in drastic disorder in the body. Cholesterol is implicated in several diseases, particularly those prevalent in modern society such as atherosclerosis and Alzheimer’s disease. While it is true that nutrition is a significant risk factor for many diseases involving cholesterol (particularly atherosclerosis), it is also important not to neglect potential disorders on a molecular level. At the elementary level, all cholesterol (as well as lipid metabolism in general) disorders are categorized either as hyperlipidemia or hypolipidemia, depending on which direction the dysregulation is exhibited (7, 8).

### Hypolipidemia

Hypolipidemia is only rarely observed clinically. While this does not necessarily imply low prevalence in the population, it suggests that most cases are asymptomatic (as are those who are discovered in routine lipid screenings). While genetic disorders exist, which directly cause hypolipidemia (abetalipoproteinemia, hypobetalipoproteinemia, and chylomicron retention disease), it is most often observed as a symptom of a mostly unrelated systemic disorder, such as chronic inflammation, acute or chronic infection, undernutrition and hyperthyroidism among others (9).

### Hyperlipidemia

Hyperlipidemia, also referred to as hypercholesterolemia or simply “high blood cholesterol,” has shown an increasing incidence as well as prevalence in modern society. It can be classified as either primary (familial) or secondary (acquired), depending on the underlying causes. While the primary variant shows a relatively stable incidence relative to total population size, secondary hyperlipidemia incidence has increased over the course of recent decades (7).

Most cases of primary hyperlipidemia can be traced back to mutations in LDLR, apolipoprotein B, or proprotein convertase subtilisin/kexin type 9 (10, 11). Secondary hyperlipidemia on the other hand is usually associated with a high calorie diet or other metabolic disorders, such as diabetes. The health consequences of hyperlipidemia can potentially be life-threatening. The most commonly known consequence is without a doubt the accumulation of fatty material in the form of xanthomas on the lining of blood vessels. If left unchecked, this can eventually cause blockage of the blood vessel and thus heart attacks or stroke. There are other consequences, however, which are not as obvious. These more insidious malignancies are usually tied to the specific impact of cholesterol on the function of cells. The impact on T cells will be discussed in this review.

## Cholesterol Metabolism in T Cells

Considering that cholesterol is important in maintaining cell membrane stiffness, its importance to immune cells, one of the cell types relying the most on motility and membrane–membrane interactions with other cells, is easy to see. Immune cells need to be able to move from tissue to tissue, need to be able to form a synapse with other cells, and sometimes even engulf target cells. While cholesterol is thus important for all subsets of immune cells, its importance to T cells will be examined here.

Since cholesterol can also be considered a kind of nutrient for the cell, it is first important to talk about how T cells manage their nutrients. Usually, a cell would derive energy from glycolysis, and oxidative phosphorylation, depending on how much oxygen is available to them. In highly proliferative cells, however, a much higher priority is placed on glycolysis (12, 13). One can observe this not only in proliferating T cells but also as the Warburg effect in cancer cells. Since proliferation requires lipids for membrane synthesis, fatty acid catabolic pathways are mostly suppressed in proliferative cells. In contrast, the mechanisms of fatty acid biosynthesis as well as cholesterol synthesis are strongly upregulated in T-cells (13).

Results regarding the cholesterol metabolism in different T cell subsets have so far been inconclusive. In one study (14), higher levels of GM1 were measured in thymic CD8^+^ single positive T cells when compared to CD4^+^ single positive T cells and CD4^+^CD8^+^ double positive T cells. Splenic CD8^+^ T cells had higher levels of GM1 and total cholesterol than CD4^+^ T cells. No obvious difference of GM1 expression between naive and memory T cells was noted.

### Impact of Cholesterol on T Cell Activation and Proliferation

Considering that T cell activation generally triggers a large-scale proliferation of the activated cells, anabolic pathways are critical during this process of activation. This has been well established in cancer cells, where high proliferation is the primary defining characteristic (15, 16). Treating a population of naïve T cells with Statin suppresses progression of their differentiation and cell cycle. Statin is an inhibitor of HMG-CoA reductase, the enzyme responsible for catalysis in the rate-limiting step of cholesterol biosynthesis. Furthermore, inhibition of this enzyme leads to decreased rates of DNA synthesis in many highly proliferative cell populations, such as lymphocytes, fibroblasts, and PDGF-treated smooth-muscle cells (17, 18). These downstream effects of statin treatment play a role in stagnating T cell activation. It is important to note, that not all statin-mediated changes in the cell are inherently tied to cholesterol levels. As statins inhibitory target is further upstream from cholesterol synthesis, cholesterol-independent effects may be at work as well.

Once a T cell receives an activation signal, sulfotransferase 2B1 is upregulated to reduce oxysterol level, SREBP processing is promoted, and LXR signaling is suppressed (19). Induction of LXR signaling leads to stagnating proliferation, while a knockout confers a proliferative advantage. This link of LXR signaling to proliferation is directly related to cholesterol transport, as inactivation of the transporter proteins (e.g., ABCG1) prevents the effect of LXR induction. This showcases the importance of LXR as a metabolic checkpoint in T cell activation and immune cell function in general (19–21). SREBP has been shown to be indispensable for CD8^+^ T cell activation and proliferation as well (21). Metabolism checkpoints for T cell activation and proliferation can be found in many of the subset populations and include not only lipid metabolism but also glycolysis. mTOR and TRAF6, for example, have been shown to impact the differentiation of CD8^+^ T cells *via* their downstream targets, regulating cell metabolism (22–26). Other important regulatory proteins, such as Myc and AMPK, have been implicated as well (27–29).

There is some evidence to suggest that the role of lipid metabolism may not be as simple as a binary toggle for proliferation. Since the rate of *de novo* fatty acid synthesis can impact differentiation either to T_h_17 or T_reg_ cells (30), a complex underlying regulatory system is implied.

The importance of understanding this relationship becomes clear when looking at cancer cases. The inability of T cells to cope with a developing tumor is aggravated as the energy consuming tumor decreases available nutrients in the body. If it reaches a point where blood LDL and HDL levels drop, even initial T cell activation can be compromised (31).

Exogenous cholesterol levels can shift the T cell population balance on the level of an entire organ in the body, as was observed in increased T_reg_ differentiation in hypercholesterolemic conditions in the liver (32).

### Lipid Rafts, Membrane Dynamics, and Nanoclustering

Since the membrane is mostly made up of lipids, their dynamics impact the function of embedded proteins (33–35). Changing the charge of acidic phospholipids, for example, can directly alter TCR and CD28 activation (36–38).

These findings suggest one of the primary mechanisms by which cholesterol can change T cell activation: *via* changing dynamics of lipid rafts and the membrane in general and therefore increasing or decreasing the colocalization of crucial receptors. Lipid rafts can be categorized as heterogenous regions of lipid distribution across the membrane, which are distinct in their composition and fluidity. They represent one of the corralling mechanisms active in the cell membrane, since they allow for spatial control of membrane-associated proteins. The importance of lipid rafts, as well as their dependence on cholesterol concentration is well known (39–42). The immunological synapse has been considered a physiological form of a “lipid raft” (43). Using Miltefosine to disrupt normal lipid raft dynamics impacts T cell proliferation immensely, showing an over 50% reduced rate of proliferation.

Spatial control over receptors is especially crucial for receptors reliant on colocalization to achieve their active conformation (44). These receptors need to bind partner molecules and stay in stable association to function. Introducing a factor which promotes either the bound or unbound state modulates the overall sensitivity of the receptor. This kind of control over receptor clustering was shown to modulate sensitivity independent of the associated ligand (45). Maintaining precise spatial control over receptor nanoclustering partially determines sensitivity to external stimuli, as is the case for CD4 and the TCR (46). Lipids in general (and cholesterol in particular) can modulate receptor signaling *via* promotion of various conformational states of membrane receptors (37).

Not only colocalization of receptors on the membrane at large is implicated in this mechanism. Membrane fluidity and spatial control of receptors is crucial in maintaining one of the main avenues of communication for immune cells: the immunological synapse (47), since many of the involved proteins have to remain in close association.

### Cholesterol As Signaling Molecule in T Cells

Apart from modifying membrane dynamics, cholesterol influences cellular signaling as direct ligand as well. Synthetic agonists to the LXR receptor family were shown to mediate an anti-inflammatory response in macrophages and other cells of the immune system (48, 49). The importance of these regulators of lipid metabolism to the innate immune system in general has been shown as well (50–53).

There are also cases, where it is still unclear whether the involved mechanism is related to direct cholesterol binding, or whether it is a downstream effect of the previously discussed modulation of membrane dynamics. Administration of squalene, a cholesterol precursor, was shown to increase the resting population of CD4 T helper cells as well as predispose T cells toward higher inflammatory reactivity (54) and direct depletion of cholesterol in the growth medium of T cells as well as diet of mice led to reduced rates of T cell activation and proliferation (55). It has been previously shown that rapidly dividing cell populations react to a higher demand for cholesterol with increased scavenging of serum LDL mediated *via* an increase of the responsible receptors (56).

**Figure 1:**
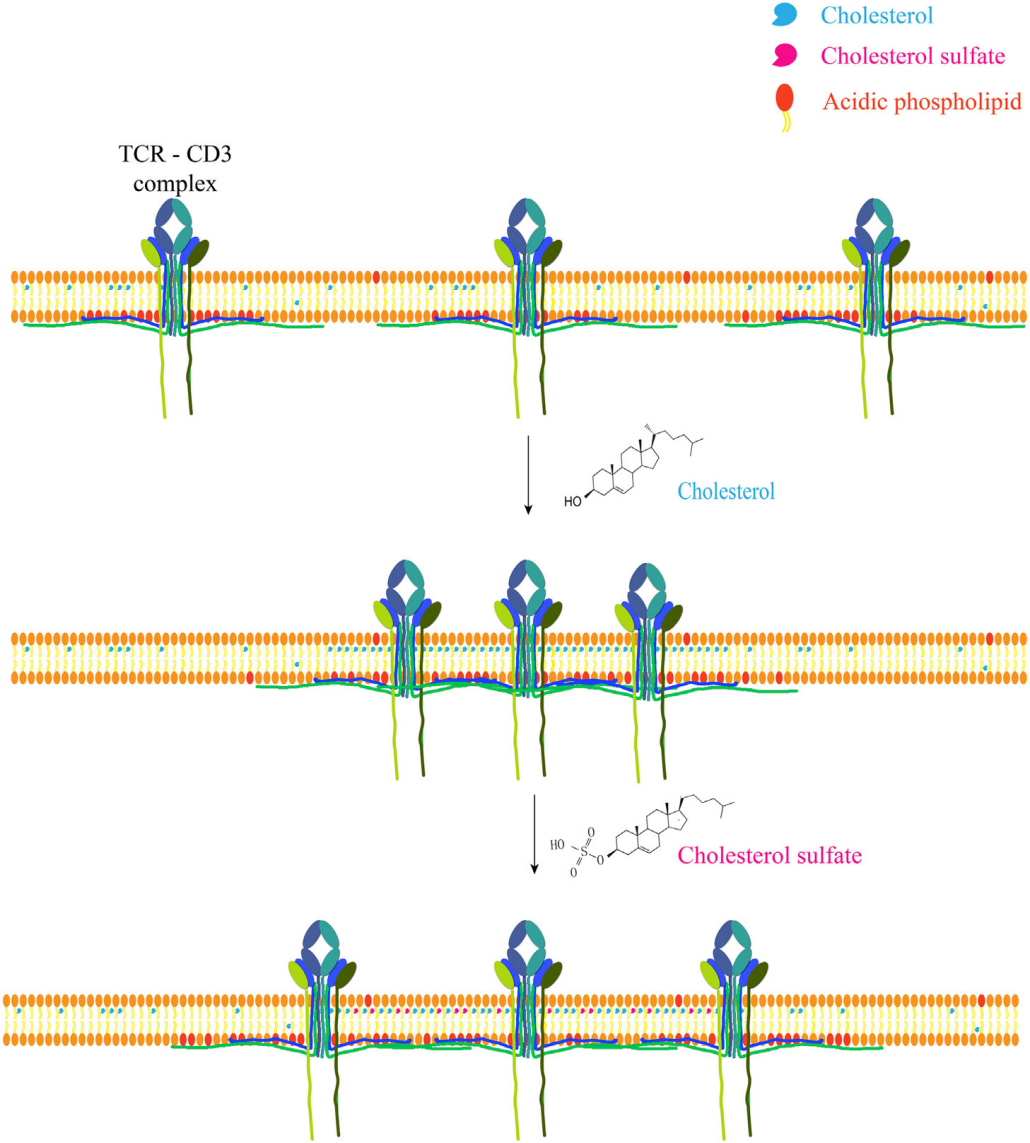
Cholesterol and metabolite regulate T cell receptor clustering. Cholesterol can directly bind to the transmembrane domain of TCR-β chain to mediate TCR clustering on T cell surface, which can increase the avidity of TCR to foreign antigens and therefore augment TCR signaling. Meanwhile, cholesterol can keep TCR at an inactive conformation to avoid spontaneous signaling. Another safety control mechanism of TCR activity is the sequestration of TCR signaling motifs within the membrane bilayer *via* the ionic interactions between acidic phospholipids and polybasic regions in CD3 cytoplasmic domains. Cholesterol sulfate, a negatively charged cholesterol metabolite, however, can disrupt TCR clustering *via* the interference of cholesterol–TCR interaction. The underlying mechanism remains to be further illustrated.

Recently, the direct ligand role has gained some more momentum, however. Actual direct cholesterol binding modifies TCR *via* allosteric signaling (57, 58) and a direct binding motif for cholesterol has been discovered and is present in a number of membrane proteins, such as the metabotropic glutamate receptor, adenosine receptor A1, and GABA type B receptor (59). Cholesteryl esters play a role as conformational stabilizers (or cofactor) in CD1c, allowing self-reactive T cells to bind the protein (60). Cholesterol sulfate (structurally similar to cholesterol) can disrupt TCR multimers, effectively inhibiting signal transduction. This most likely occurs due to displacing receptor bound cholesterol (61) (Figure 1).

## Modulating T Cell Function *via* the Cholesterol Pathway

Based on the demonstrated importance of cholesterol and its related regulatory proteins, it is easy to see the potential of some of these components as treatment targets. Potential applications as well as methods for making use of them will be discussed in the following.

### Tools for Modulation

Statins are, of course, the most obvious and ubiquitous choice of drug intervention for modulating cholesterol metabolism. They have been widely used in atherosclerosis treatment for many years. While their actual effect has come under increasing scrutiny in recent years (62), statins remain an effective HMG-CoA reductase inhibitor. Atorvastatin can normalize T cell signaling and reduce the production of IL-10 and IL 6 by inhibiting cholesterol biosynthesis and reducing cholesterol levels in the T cell membrane (42). As a cholesterol-depleting reagent, methyl-β-cyclodextrin is used to disrupt lipid rafts and prevent their clustering, thus abrogating the heightened proximal TCR signaling and reversing the hyperactivity of T cells (63, 64). Synthetic agonists specific to a particular subset of the LXR receptor family have already demonstrated their effectiveness (65). By now, many drugs which target LXR specifically are available (66). The impact on T cells has also been shown with decreased cell proliferation and colony formation rates (67). Treatment options also include the precursors of cholesterol in the biosynthesis pathway, some of which have more favorable qualities in terms of bioavailability. Squalene treatment increases resting CD4 T cell population and desmosterol functions as an effective LXR agonist and SREBP repressor (54, 68).

As most signaling of T cells is mediated *via* surface proteins, these proteins form a very attractive target for modulation. There have already been studies trying to modulate T cell function *via* these receptors (69) in traditional, antibody-based ways. Modulating the receptor clustering to increase receptor sensitivity, however, is a promising approach for modulation without the need of expensive antibodies or other vehicles. For example, small molecule-mediated inhibition of ACAT1 can cause better TCR clustering in CD8 T cells (44).

### Potential Treatment Applications

Metabolism has long been a target for cancer treatment (70). Usually, the treatments target the metabolism of cancer cells to deprive them of nutrients. Targeting the metabolism of antitumor immune cells instead may provide new treatment options. It is clear that immune cells undergo significant changes in the tumor microenvironment. Some of these changes may be mediated by metabolism signaling (71, 72). ACAT1 inhibition, leading to higher levels of plasma membrane cholesterol, induces higher antitumor activity in CD8 T cells (44) *via* enhanced receptor clustering on the cell surface. Under the same principle, modulation of cholesterol levels can decrease colocalization of crucial receptors in autoimmune diseases such as systemic lupus erythematosus (42). As such, it could potentially take the role of a sliding scale for sensitivity of T cells in general. Whether it is applicable in other diseases, such as long-term treatment of atherosclerosis *via* atorvastatin to inhibit the inflammatory response of CD4 T cells remains controversial (73, 74).

Using targets in metabolism for cell modulation always carries a risk of off-target activity. Interestingly, Cre-lox-based knockout of SCAP (and therefore SREBP1 and 2) led to asymptomatic phenotypes in the observed T cell populations, as long as they remained in a quiescent state. This suggests that quiescent cells could remain largely untouched from modifying these response elements (21).

**Figure 2:**
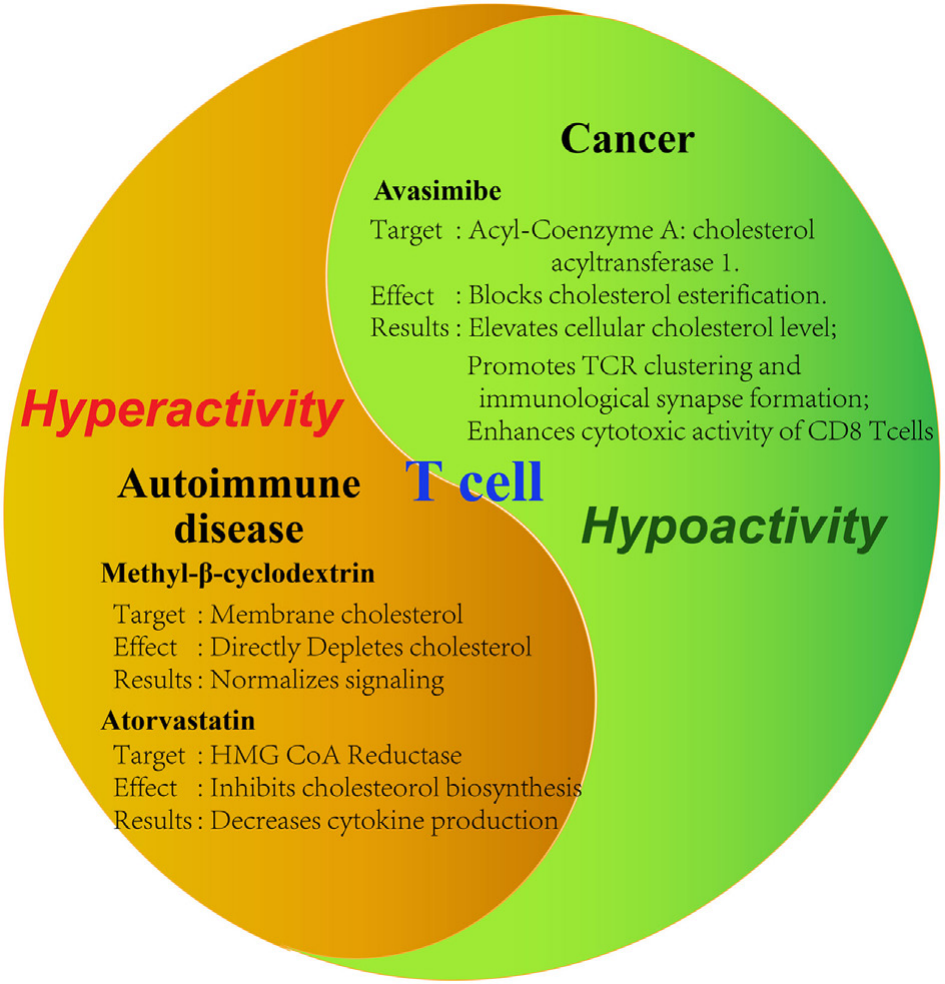
Tools for modulating cholesterol metabolism of T cells. Summary of demonstrated tools for the modulation of T cell activity by targeting cholesterol metabolism in disease contexts. Tumor-infiltrating T cells are characteristic of hypoactivity, whereas auto-reactive T cells are of hyperactivity. Therefore, different strategies should be applied to modulate cholesterol metabolism in different disease contexts. Specific reagents as well as the observed effects on the cell are summarized. Avasimibe: inhibitor of acyl-coenzyme A: cholesterol acyltransferase; GW3865: agonist of liver X receptor; methyl-β-cyclodextrin: strong cholesterol binder that can deplete membrane cholesterol; atorvastatin: inhibitor of hydroxymethylglutaryl-coenzyme A (HMG-CoA) reductase.

As has been demonstrated in numerous studies now, cholesterol is important in the activation and proliferation of T cells. From this conclusion, the thought of utilizing this for treatment of diseases arises. Initial studies examining the effect of modulating cholesterol levels on cancer and autoimmune diseases already show promising results (Figure 2). Counteracting the immunosuppressive tumor microenvironment, for example, could be an important aspect of all cancer therapies, potentially raising their chances of success across the board.

### Conclusion and Perspectives

In recent years, the physiological and pathological importance of cholesterol metabolism in T cell immunity starts to be revealed, but we are still at the early stage to fully understand its roles and regulatory mechanisms. Modulating cholesterol metabolism appears to be a promising therapeutic direction to harness T cell activity in cancer and autoimmunity. Several key questions remain to be answered in the future: 1. What are the function of various cholesterol metabolites in T cells? 2. Why do different T cell subsets have different cholesterol metabolisms and what is the functional importance of this difference? 3. In different disease contexts, how does local environment affect cholesterol metabolism of T cells and how can this process be modified to rescue T cell activity? Answering these questions can eventually lead to the development of new cholesterol-based immunotherapies against various human diseases.

## Author Contributions

CX conceived the writing. AB wrote the first version. HZ revised the paper and made the figures. MX contributed to figure discussion.

## Conflict of Interest Statement

The authors declare that the research was conducted in the absence of any commercial or financial relationships that could be construed as a potential conflict of interest.
